# Achieving COVID-19 and Routine Immunization Data Systems Integration on the Electronic Management of Immunization Data System in Nigeria

**DOI:** 10.9745/GHSP-D-23-00149

**Published:** 2024-02-20

**Authors:** Temitayo Tella-Lah, Dayo Akinleye, Abdulmumuni Samuel Aliyu, Tope Falodun, Stephanie Okpere, David Akpan, Olayinka Orefunwa, Loveth Metiboba, Judith Owoicho, Bassey Okposen, Amaka Nwabufo

**Affiliations:** a eHealth Africa, Abuja, Nigeria.; b National Primary Health Care Development Agency, Abuja, Nigeria.

## Abstract

**Background:**

In 2021, Nigeria developed a novel Electronic Management of Immunization Data (EMID) system to address COVID-19 data management challenges and ensure the successful implementation of its COVID-19 vaccine deployment plan. The EMID system was envisioned to be interoperable with the DHIS2 national data management system and serve as a gateway into the integration of other primary health care (PHC) service data management. However, the EMID system faced challenges, including inability to filter reports, missing or loss of data, and difficulties with data synchronization, which curtailed its potential to meet the country’s needs for COVID-19 data management and negatively impacted system scalability to enable integration with other PHC data systems.

**Methods:**

Multilayered stakeholder interviews were conducted to determine the optimal functionality requirements for the EMID system. Based on these findings, an optimization plan was designed and implemented to address identified gaps and create a more stable and scalable system to enable further system integrations. Following optimization, a routine immunization module was developed and integrated with the EMID system as a first step to achieving an integrated data management system for PHC services in Nigeria.

**Results:**

The integrated system currently provides an opportunity to address data fragmentation and strengthen PHC service delivery in Nigeria. By allowing 1 health care worker to deliver both vaccinations, there is also potential for reduction in cost and redundancies, informing redistribution of the health workforce and overall system strengthening.

**Conclusion:**

The journey from the initial challenges faced by the EMID system to the development of an integrated system for PHC services in Nigeria has been a transformative one. Through a thorough optimization process, training and capacity-building, stakeholder-driven improvements, and an elicitation exercise, the EMID system has evolved into a powerful tool for addressing data fragmentation and enhancing public health service delivery in the country.

## Abstract (precis)

The authors discuss approaches to transforming Nigeria’s COVID-19 data management system (the Electronic Management of Immunization Data (EMID) system) into an optimized platform for integrated primary health care data management.

## Teaser

**Key Findings:**

- The Nigeria Electronic Management of Immunization Data (EMID) system was optimized to address challenges with managing COVID-19 vaccination data, including interface issues and slow data synchronization.
- Resolving synchronization and speed issues helped facilitate the integration of a routine immunization module to meet the country’s vision of an integrated primary health care service data management platform.
- The EMID system optimization and integration of COVID-19 and routine immunization data into 1 system has resulted in a significant increase in the number of vaccinations documented, improved data synchronization, faster data entry speed, better data quality and integrity, and a streamlined health workforce to deliver COVID-19 vaccines and routine immunizations.

**Key Implications:**

- When developing country-level systems, such as the EMID system, developers could benefit from using a modular, human-centered approach that engages stakeholders from inception, conducts robust elicitation exercises that accommodate the needs of users across various levels of implementation, and incorporates feedback for better scalability.
- Digital health solutions like the EMID system confer advantages in enhancing high-quality data management for strengthened national immunization programs.

## BACKGROUND

The COVID-19 virus was declared a global pandemic in March 2020 by the World Health Organization^1^ and has had a significant impact on the world, ranging from widespread illness to death and economic disruption. To date, there have been more than 500 million confirmed cases and over 6 million deaths globally.^2^ Africa, initially thought to be less affected by the pandemic due to its relatively young population and previous experience with infectious diseases, has suffered significantly from the virus,^3^ with more than 10 million confirmed cases and over 200,000 deaths reported across the continent as of April 2023.^1^ In Nigeria, the first case of COVID-19 was reported on February 27, 2020.^4^ Since then, there have been more than 4 million confirmed cases and over 100,000 deaths in the country.^5^

The pandemic exposed weaknesses in many health systems that impacted service delivery, often disrupting access to care. Among the most impacted health services were routine immunization (RI), exposing millions of children to the risk of vaccine-preventable diseases, such as measles, polio, diphtheria, tetanus, and pertussis (DTP).^6^ In 2022, a study conducted in Nigeria reported that, before the COVID-19 pandemic, Nigeria had a coverage rate for the third dose of the DTP vaccine of 57%. Fully vaccinated children aged 12–23 months accounted for only 31% of this age group, with urban areas having a coverage rate of 44% and rural areas of 23%. The onset of the COVID-19 pandemic further exacerbated the situation, causing a drop in immunization coverage. As a result, an additional 19% of eligible children (zero-dose children) did not receive RI. This heightened the risk of vaccine-preventable disease outbreaks among children in Nigeria.^6^ Additionally, lack of access to basic health services is among the reasons why children remain vulnerable to vaccine-preventable diseases. An evaluation study conducted in a southern district of Nigeria revealed that about 12% of caregivers reported that the distance to immunization sites prevented children from being vaccinated.^7^ Furthermore, the diversion of staff to COVID-19 vaccination services limited the number of staff who were able to deliver RI.

As in many countries, measures were taken by the Nigerian government to control the spread of the COVID-19 virus, including the implementation of lockdowns, travel restrictions, and the establishment of testing and treatment centers.^8^ Several strategies, including the SCALES (Service Delivery, Communications, Accountability, Logistics, Electronic management of immunization data, and Supportive supervision) strategy, were also implemented to ramp up uptake of COVID-19 vaccines and improve data use for effective decision-making. However, COVID-19 pandemic control efforts faced significant challenges, including limited resources, inadequate infrastructure, and a lack of standardized data collection and reporting processes. The lack of quality data on COVID-19 cases and related deaths also hindered the identification of priority groups for vaccine rollout, spotlighting the need for a robust data management system to collect, store, and analyze data in a timely manner.^8^^,^^9^ Therefore, addressing the data management challenge was a crucial step to ensure Nigeria maintained equitable access to the COVID-19 vaccine. Equally important was the need to integrate COVID-19 data with other routine primary health care (PHC) service data to ensure a robust and sustainable data management system.^10^ To actualize this goal, it became necessary to develop a system that could facilitate the overall management of immunization data and other programs at PHC facilities in Nigeria.

In 2021, Nigeria developed the novel Electronic Management of Immunization Data (EMID) system to address COVID-19 data management challenges and ensure the successful implementation of its COVID-19 vaccine deployment plan. Designed to be interoperable with the DHIS2 national system, the EMID system was developed for managing COVID-19 vaccination data in Nigeria and ultimately facilitating the overall management of immunization data and other PHC service data, including vaccination scheduling, real-time client data entry, collation and analysis, and validating data accuracy. However, the EMID system faced challenges, including the inability to filter reports, missing or loss of data, and difficulties with data synchronization, which curtailed its potential to fulfill the country’s needs and negatively impacted the system’s scalability and the integration of other PHC data. This necessitated the need for optimization of the existing EMID system to meet the country’s needs. We articulate the processes undertaken to develop an optimized EMID system integrated with an RI module, achievements recorded so far, as well as lessons learned and key recommendations that may be useful in the design and implementation of similar programs.

Nigeria developed the novel EMID system to address COVID-19 data management challenges and ensure successful implementation of its COVID-19 vaccine deployment plan.

## EMID SYSTEM OPTIMIZATION PROCESS

The EMID system consists of the following 4 tools.

1. The EMID mobile application is used by the recorders to collect clients’ data at the point of vaccination.
2. The DHIS2 Web application is used for registration, reporting, and visualization of vaccination data. It doubles as a central EMID tool, as all other applications fetch or submit data to the platform.
3. Public Registration (PubReg) is a self-registration portal used by the public to schedule vaccination appointments before visiting the vaccination sites.
4. The verification portal helps to generate and verify client vaccination information using Quick Response code technology.

**FIGURE 1:**
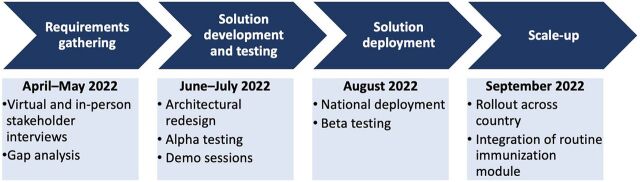
Four Key Phases of the Electronic Management of Immunization Data System Optimization Process

During the program initiation phase, 4 key phases were identified as being essential to successfully optimizing the EMID system: requirements gathering and needs assessment; solution development and testing; solution deployment; and scale-up. Figure 1 shows the timeline associated with the 4 phases and the activities undertaken in each phase. The relationship between these phases was sequential, with each phase building upon the previous one. The requirements gathering and needs assessment laid the foundation for the optimization of the EMID system. The solution development and testing ensured that the system was redesigned and optimized to meet the optimal functionality requirements, while the deployment phase put the system into use across the 36 states and the Federal Capital Territory (FCT) in Nigeria through an iterative process with continuous improvements to the system based on user feedback. Finally, the scale-up involved the development and integration of an RI module to facilitate the achievement of an integrated COVID-19 and PHC RI service delivery data management system.

### Requirements Gathering and Needs Assessment

A requirements gathering and needs assessment was conducted to establish the functionality requirements for the EMID system. The functionality requirement is related to the existing data collection system in the country. This involved qualitative stakeholder interviews with national and state governments, technical partners, and the relevant authorities for data management in Nigeria.

Over the course of 1 month, we employed a physical random sampling method to select and conduct in-person and virtual interviews (Table 1). A preliminary discussion was done with the Nigerian National Primary Healthcare Development Agency (NPHCDA) Information and Communications Technology team, who are the custodians of the EMID system, to understand the most commonly reported and/or experienced challenges associated with the system. Findings from the preliminary discussions were then used to inform the design of questionnaires for both the virtual and in-person interviews. Virtual interviews were conducted with a total of 160 respondents across the country. The virtual interviews were useful for testing the questionnaires and getting basic insights regarding using the EMID system. The collated findings were also used to refine the questionnaires for the in-person interviews.

**TABLE 1.** Electronic Management of Immunization Data System Virtual Interview Respondents Disaggregation

**Stratification**	**Target**	**Total Persons Interviewed **
National	At least 1 key contact personnel from the 4 partner organizations: (eHealth 4 Everyone, Nigerian National Primary Healthcare Development Agency, Health Information Systems Program, and Electronic Management of Immunization Data app developers)	8
Zonal	6 Nigerian National Primary Healthcare Development Agency personnel per zone	36
State	1–2 state designated officers	40
Local government area	2 local government area officers per state and 2 recorders	76
Total		160

**FIGURE 2:**
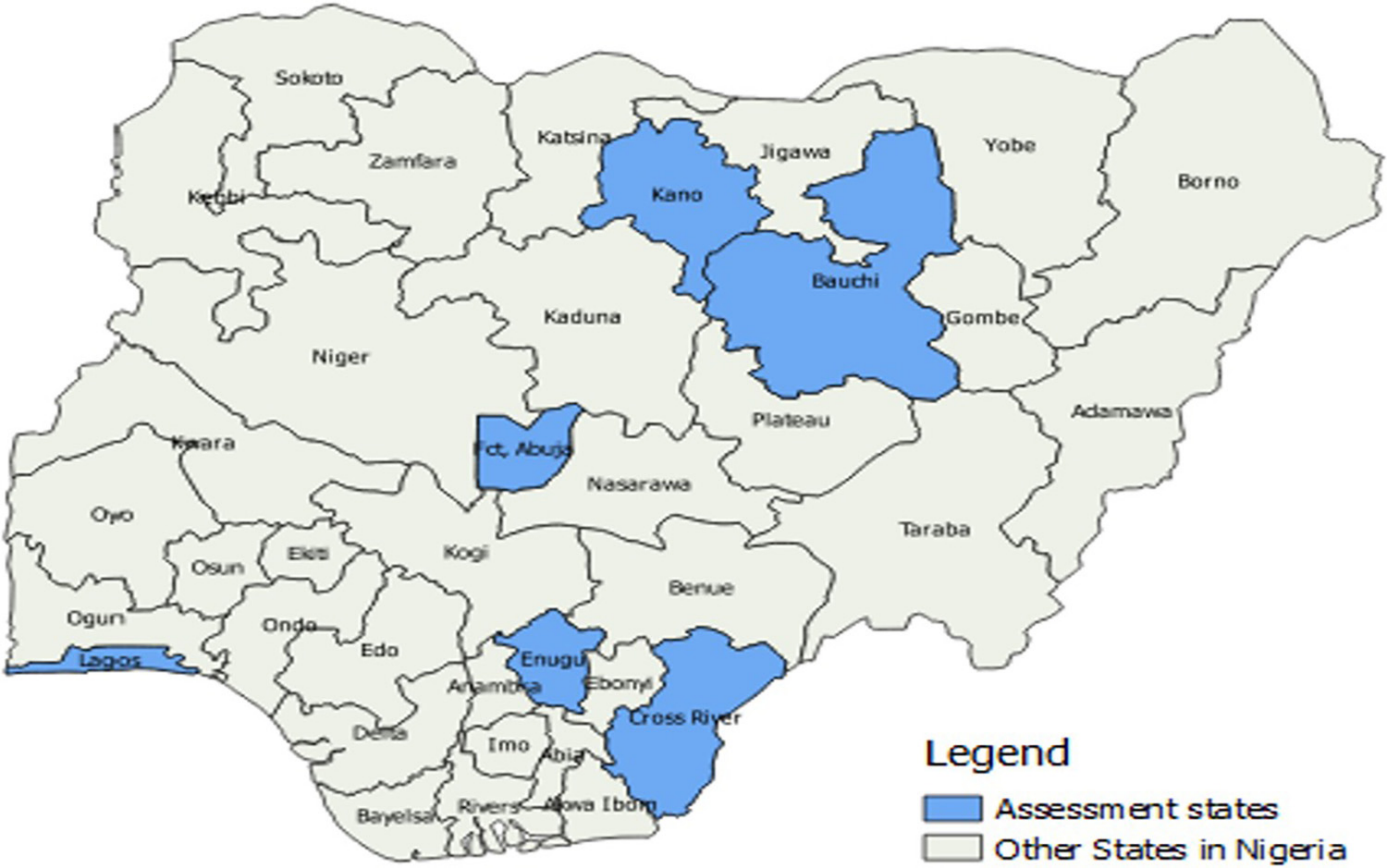
Map of Selected States in Nigeria Where In-Person Interviews Were Conducted During the Requirements Gathering Phase

The in-person interviews were conducted with EMID system users (vaccinators, recorders, and state and local government area [LGA] EMID focal persons) across 6 states in Nigeria. Nigeria is divided into 6 geopolitical zones: an administrative grouping of the country’s 36 states and the FCT based largely on geographic locations and similar ethnicities and political history. One state per geopolitical zone was selected. The in-person interviews allowed in-depth interviews with the respondents and enabled us to confirm and/or assess all identified and unidentified issues physically at selected health facilities across the assessment states. In-person interviews were conducted with a total of 59 respondents spanning across the 6 selected states: Bauchi, Cross-River, Enugu, FCT, Kano, and Lagos, each representing 1 geopolitical zone (Figure 2). Respondents were selected across both rural and urban settings in each state and included EMID recorders (supersites and other sites) and EMID focal persons at the state, LGA, and national levels (Table 2). Data were collected without the intention of establishing generalizable research and primarily focused on outcomes related to developing this data management tool.

**TABLE 2.** Electronic Management of Immunization Data System In-Person Interview Respondent Disaggregation

	**No.** **(n=59)**
Location	
Urban	19
Rural	13
Hard-to-reach	4
User type	
Recorder	
Other facilities	31
Supersite	13
Electronic Management of Immunization Data system focal person	
State	6
Local government area	5
National	4

Interview questionnaires asked participants about the functionalities of the existing EMID system and about what they viewed as optimal functionality features, including what roles and permissions were necessary. A technical assessment of the EMID system architecture was also conducted by reviewing the existing architectural design, database model, and code structure to assess the existence of any technical deficiencies in the system and the EMID system’s alignment with software standards. We also conducted stress tests, load tests, and application programming interface integration testing to test the software infrastructure of the existing system.

Our assessment revealed multiple challenges with the existing EMID system: problems with the user interface (e.g., inaccurate drop-down options, fields not aligned in tab sequence, and irrelevant graphics); slow data synchronization to the point of dysfunction; frequent loss of data across the different database sections; and inability to adapt the code underlying the overall system or add features to the database.

**FIGURE 3:**
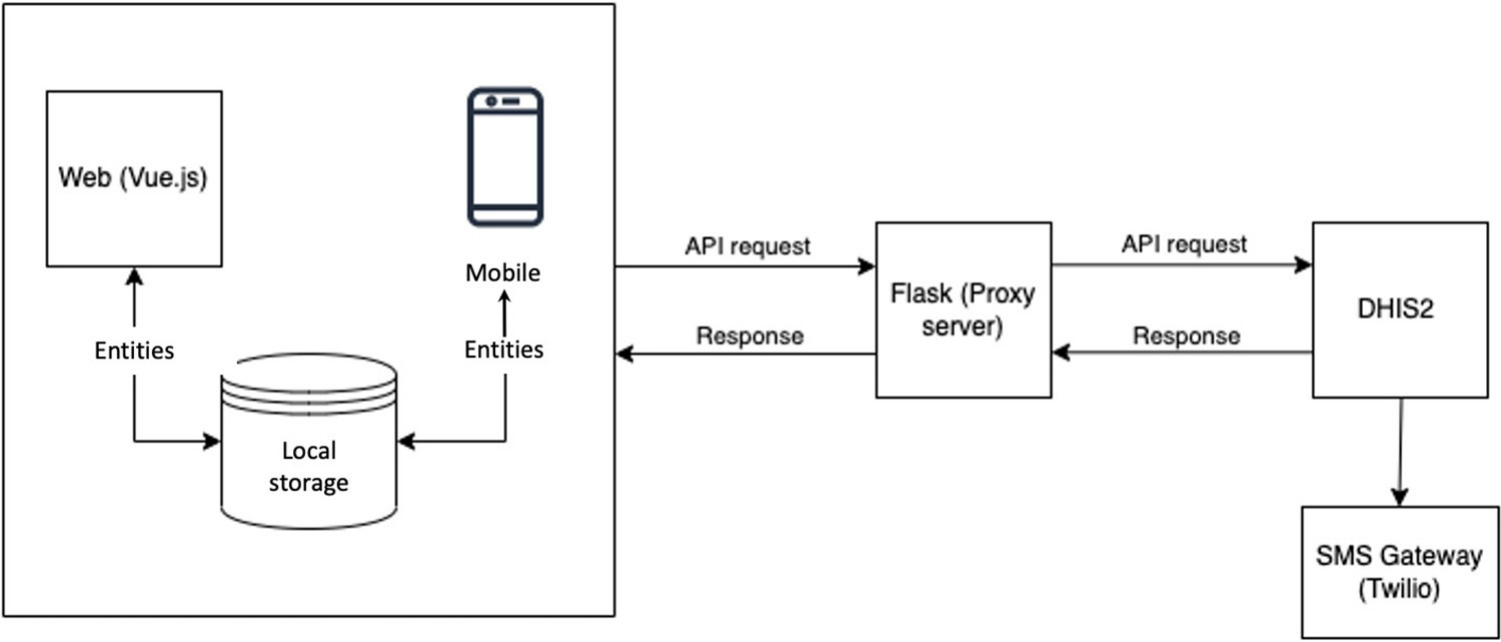
Electronic Management of Immunization Data System Baseline Architecture Abbreviations: API, application programming interface; SMS, short message service.

Although the existing EMID system was interoperable with DHIS2, the interaction was not seamless. A primary issue in the initial system was a proxy server placed between the EMID system and DHIS2 that consistently experienced failures during peak hours, severely hampering communication between the EMID system and DHIS2 (Figure 3). The findings from the assessments and technical review gave rise to the gap analysis detailed in Table 3.

**TABLE 3.** Electronic Management of Immunization Data System Gap Analysis

**Baseline Findings**	**Gap Identified**
**Electronic Management of Immunization Data Mobile Application**	
Data synchronization issues and missing data on EMID application.	Existence of a proxy server between the EMID mobile application and DHIS2 server created an extra layer therefore slowing down the synchronization process and impairing the functionality of the EMID application.
	SMS functionality for online registration was not functioning.
Hard-coded components of EMID system were out of date.	Existing hard code did not allow modification and integration of new features.
Poor user interface and experience.	Functionality of the database was not conducive to a productive workflow and users became frustrated with the system.
**PubReg**	
Updates on the PubReg were carried out on directly on the database.	This made updating the platform difficult and time consuming.
PubReg was enabled to only perform single registration for individual vaccinations.	There was no option for users to create bulk/group vaccination registrations to cater for multiple registrations by corporate organizations.
No user account interface.	Client information and/or vaccination schedules could not be edited/changed once registered on the platform.
PubReg and verification portals were maintained as separate platforms.	The existence of these parallel systems resulted in data duplication.
**Verification Portal**	
Updates on the verification portal were carried out on the backend.	Updating the platform is difficult and time-consuming.
Low level of security.	There was a need to better authenticate accounts.

Abbreviations: EMID, Electronic Management of Immunization Data; PubReg, Public Registration; SMS, short message service.

### Solution Development and Testing

**FIGURE 4:**
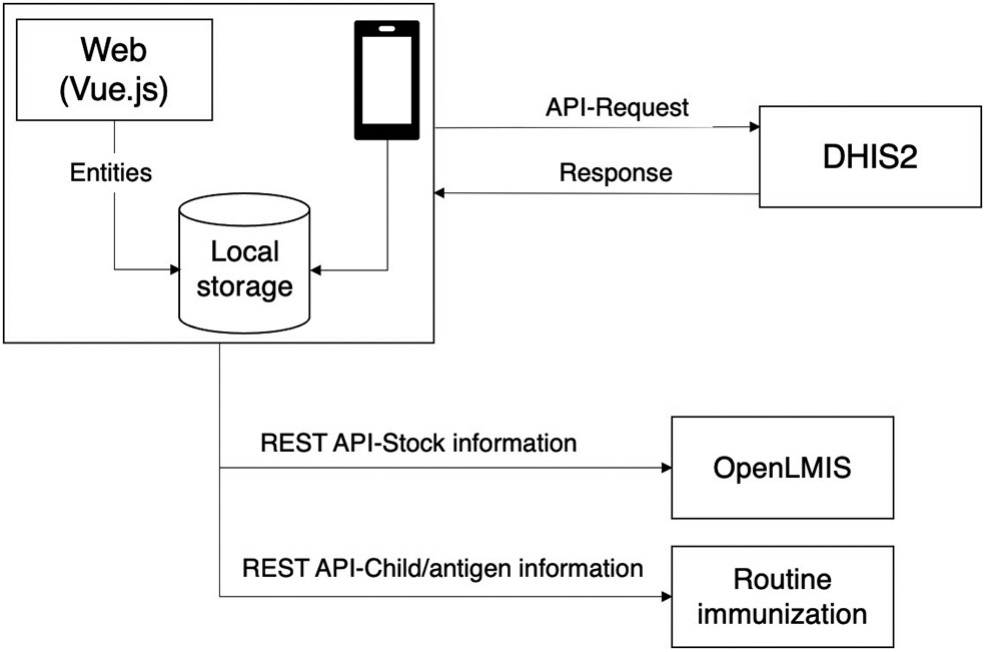
Electronic Management of Immunization Data System Redesigned Architecture Abbreviations: API, application programming interface; EMID, Electronic Management of Immunization Data; LMIS, logistics management information system.

The development and testing phase aimed to address the problems highlighted in the previous phase. First, the architecture of the existing EMID application was redesigned to eliminate the proxy server, enabling direct communication between the EMID system and DHIS2 (Figure 4). Extensive functional and nonfunctional tests were then performed and improvements and refinements were made to the system after each testing iteration. This not only resolved synchronization and speed-related issues but also facilitated the seamless integration of additional modules (e.g., RI) into the application without negatively impacting EMID system performance and speed. This phase also included restructuring EMID application code, which resolved data synchronization and hard code issues presented in the gap analysis. It also enabled automatic synchronization of records without manual prompting. A redesign of the application interface and logic was also done. This was geared toward addressing the poor user interface gap and improving the user experience.

In addition, the existing EMID application was restructured using progressive web application technology to enhance its versatility across multiple devices and systems. Throughout the development process, rigorous testing was conducted to ensure proper functionality of the optimized system. Demonstration sessions were also done with the NPHCDA team for feedback to inform iterations in the development process before deployment.

### Solution Deployment

After the development and testing phases were finalized, a deployment process was executed in which the optimized EMID application was deployed to the Google Play Store, making it available for use across the 36 states and the FCT in Nigeria. Support was extended to the vaccination teams across states to help them update their application to the optimized version. To facilitate the adoption of the optimized application, an instructional video was developed to train the EMID system users on new features and how to navigate the application in line with the changes made to the application logic. The instructional videos and documented guides were shared with the NPHCDA for dissemination to vaccination recorders and relevant EMID system state teams across all states in the country. The NPHCDA team shared the video with relevant focal persons and EMID system users across the state, LGA, and facility levels using the WhatsApp platform. Before the deployment, a national training of trainers was also conducted for the NPHCDA technical team to position them to resolve any issues that may be raised by users on the use of the optimized application. Virtual training sessions were also conducted across some states that had recorded low vaccination performance associated with recruiting new recorders in those states. The training took participants through the process of resolving issues related to the restructured application. Feedback and information gathered through this training informed the documentation and development of a frequently asked questions guide that was later harmonized and shared with all the state and local government focal persons.

### Scale-Up

#### Integration of RI Module

In line with the country’s vision for integration of all PHC service data on the EMID system, the next phase of the project involved the development and integration of an RI module on the EMID using an agile methodology. Similar to the optimization of the EMID system for COVID-19, the RI module integration commenced with a requirements gathering with the National Emergency Routine Immunization Coordination Centre (NERICC) team. This was followed by the development of an RI module prototype and the conducting of demonstration sessions with the team. Discussions with the DHIS2 team provided more information about the requirements necessary to optimize the RI module. The RI module for the EMID was developed within 2 months, including unit testing and integration testing with DHIS2. Two demonstration sessions were held with the NERICC team, and the module was approved for piloting after the second iteration process. The RI module was deployed to the Google Play Store on December 11, 2022.

#### RI Module Pilot

The EMID RI module has been piloted in 2 LGAs each in 2 states—Bebeji and Kano Municipal LGAs in Kano state and Ibadan Southwest and Egbeda LGAs in Oyo state. Before the pilot exercise, national training of trainers was conducted for the NERICC team and RI providers who were the major national facilitators. This was followed by LGA-level cascade training. The RI module pilot lasted 5 months (December 2022–May 2023). Some important insights have been gained from the pilot. For example, the NERICC team identified a challenge where users could only filter by name, which proved cumbersome due to the occurrence of multiple children sharing the same name. To address this, we implemented a search system to use caregiver phone numbers and vaccination IDs, effectively resolving the issue.

## EMID SYSTEM ACHIEVEMENTS

After the optimized EMID system was deployed in September 2022, a significant increase in the use of the EMID system was recorded. Documented vaccinations increased from 168,265 in August 2022 to 530,060 in September 2022 and 2,657,038 in October 2022. The increase in vaccination records during this period also coincided with the use of the optimized system for the third iteration of the SCALES strategy (SCALES 3.0). Missing data issues were also eliminated. Where network challenges hindered automatic synchronization, all unsynchronized data remained in the offline section of the application for automatic synchronization when Internet connectivity was restored rather than disappear as experienced with the earlier version of the application. The average time for recording a client’s data on the optimized system was also reduced from 3–5 minutes per client to an average of 2 minutes due to the improved user interface/logic.

After the optimized EMID system was deployed, there was a significant increase in the number of documented vaccinations.

Before the EMID-RI integration, RI data collection across the country had been primarily paper based and characterized by poor data quality and integrity with discrepancies in reported immunization coverage data, thereby limiting data-driven decision-making. The integrated system currently tracks vaccination delivery across COVID-19 and RI services, providing an opportunity to address data fragmentation and strengthen PHC service delivery in Nigeria. Between December 2022 and May 2023, the data of a total of 39,932 children (20,191 in Kano and 19,732 in Oyo) who received an RI vaccine have been recorded using the integrated EMID system across the 4 LGAs in the 2 pilot states.

The capacity of a total of 263 health workers and 46 state officers comprising LGA officers and partners across the 2 states has also been built on using the optimized EMID system (Table 4).

**TABLE 4.** Health Workers Trained on the Optimized Electronic Management of Immunization Data System

**Trainee cadre**	**Kano**		**Oyo**	
	**Bebeji**	**Kano Municipal**	**Ibadan Southwest**	**Egbeda**
Health workers	48	52	63	100
State officers	26		20	

With the integration of the RI module on EMID, the existing health workforce at the national and state levels can be leveraged for the delivery of both COVID-19 and RI vaccines. By allowing both vaccinations to be delivered by the same health care worker, there is potential for reductions in cost and redundancies, which could inform the redistribution of the health workforce and overall system strengthening in the longer term. The implementation of the performance-based measures on the integrated EMID system has also resulted in significant cost savings for the country. The integrated system can triangulate the vaccination coverage rate with workforce capacity to accurately inform human and financial resource reallocations.

## LESSONS LEARNED

For country-level systems, such as the EMID system, a robust elicitation exercise that accommodates the needs across various levels of implementation is crucial. The pandemic necessitated an urgency in the development of a data management system to respond to immediate needs; however, expanded consultation is critical in the development of a country-level system to ensure suitability for purpose, stability, scalability, and long-term sustainability. It is important that all technical stakeholders have adequate administrative access to facilitate seamless and successful systems integration and interoperability. Where more than 1 partner is involved in the development and integration process, limited administrative access and lack of clarity on goals, as well as specific roles and responsibilities, significantly slows down the process. Government buy-in, as well as the support from relevant national technical working groups and partner groups, was central to the successful development and deployment of the optimized EMID system.

Knowledge and capacity gaps among EMID users, many of whom are part-time health care workers with limited digital tool experience, significantly hampered effective application utilization. This was especially pronounced in rural areas, where users often misattributed their difficulties to application faults. The urgent recruitment of these temporary staff during the pandemic led to high attrition rates when they received permanent job offers, causing knowledge and capacity loss without proper handover. Consequently, it is essential to provide ongoing training and retraining for EMID users to maintain application proficiency. Continuous supportive supervision is also necessary and provides opportunities for on-the-job, hands-on mentoring as well as prompt issue resolution.

The design and implementation of a change management approach is important from the outset to ensure an effective and seamless transition. Adequate time and financial resources should also be allocated for user acceptance testing and application pilot before wide-scale deployment. Lastly, the design and proper definition of data security and governance frameworks for data management systems should be conducted from the outset.

### Recommendations

- Further expansion and scale-up the EMID system should be prioritized to achieve the desired integration and management of PHC service data.
- Additional modules for polio supplementary immunization activities, non-polio supplementary immunization activities, and adverse effects following immunization reporting should be considered in the next scale-up phase for the EMID system to expand its capabilities and coverage.
- Opportunities exist to leverage the EMID system for improved logistics and supply chain data management. Integration of a logistics module on the EMID system could help achieve end-end vaccine visibility and traceability, which will address a critical gap in the current system in Nigeria.
- The possibility of extending the use of the EMID system to various other public health programs beyond PHC, such as malaria, HIV, and neglected tropical diseases should be explored. This could be valuable not only in Nigeria but also in other countries.

### Strengths

The optimization and development process were driven by feedback from stakeholder-wide consultations, giving better credibility to the process. The iterative development process using an agile methodology also allowed for improvements to the system incorporating reviews and feedback from stakeholders.

### Limitations

The needs assessment and requirements gathering for the development of the EMID RI module was only conducted at the national level due to time constraints. The development process could have benefited from further consultations at the state level to ensure robustness. Also, the EMID RI module pilot is still ongoing. Further reviews and evaluations will be required after the full deployment of the EMID RI module across all states to assess and document challenges and/or successes of the integration.

## CONCLUSION

By implementing the EMID system, Nigeria has introduced an innovative strategy for improving PHC immunization data and leveraging these insights for better decision-making and improvements in health care systems performance. Like other complex health initiatives, the long-term success of the EMID system relies on the degree to which health care professionals and program managers embrace and take ownership of the new tools and protocols. Moreover, optimization of the EMID has improved the electronic medical record system in Nigeria, moving away from only reporting COVID-19 vaccination to capturing RI. Additionally, policymakers must be willing to commit resources to the necessary infrastructure and the continuous development of the essential human resources that are central to the EMID system.

## Notes

**Peer Reviewed**

**First published online:** December 22, 2023.
